# Acute pancreatitis in 60 Iranian children: do pediatricians follow the new guidelines in diagnosis and management of acute pancreatitis?

**DOI:** 10.1186/s12887-022-03509-6

**Published:** 2022-07-29

**Authors:** Mahsa Soti Khiabani, Mahya Sadat Mohammadi, Seyyed Amirreza Ghoreyshi, Pejman Rohani, Hosein Alimadad, Mohammad Hassan Sohoul

**Affiliations:** 1 Department of Pediatrics, Tehran University of Medical Sciences, Tehran, Iran; 2 Pediatric Gastroenterology and Hepatology Research Center, Tehran University of Medical Sciences, Tehran, Iran; 3 Children’s Medical Center, Tehran University of Medical Sciences, Tehran, Iran; 4 Student Research Committee, Department of Clinical Nutrition and Dietetics, Faculty of Nutrition and Food Technology, Shahid Beheshti University of Medical Sciences, Tehran, Iran

**Keywords:** Pancreatitis, Pediatric, Etiology, Fluid management, Antibiotic, Imaging, COVID-19

## Abstract

**Background:**

The incidence of acute pancreatitis in children is increasing, but causes and diagnostic and therapeutic methods are various in different centers. The aim of this study was to investigate the common causes and routine diagnostic and therapeutic methods of acute pancreatitis in children in a pediatric gastrointestinal referral center and its accordance with existing guidelines.

**Methods:**

In this retrospective, cross-sectional study, a total of 60 children with a diagnosis of acute pancreatitis, were studied.

**Results:**

The most common causes of acute pancreatitis were systemic and metabolic diseases and medications. CT scan was performed for 36% of patients, but 31% of patients, for whom a CT scan was performed had no clear indication of CT scan. Only half of the patients received fluid 1.5 times their maintenance in the first 24 h. Antibiotic therapy was performed for 48% of patients but medical indications for antibiotic treatment were found in only 34% of cases. During the COVID-19 pandemic, the relative incidence of acute pancreatitis was increased.

**Conclusions:**

In children with systemic and metabolic disease and using anticonvulsant drugs, it is important to consider the incidence of this disease. In clinical education, the risks of radiation due to unnecessary CT scans and inappropriate prescription of antibiotics need to be emphasized. More research should be done to study the association between COVID-19 and acute pancreatitis.

## Background

The incidence of acute pancreatitis, as the most common pancreatic disorder in children, has increased in recent decade [1–3]. In children, the most common etiologies acute pancreatitis include disorders or obstructions of the biliary system, impenetrable abdominal injuries, multisystem diseases, metabolic diseases, drug poisoning, and genetic predisposing factors [4–7]. Moreover, the most common systemic diseases associated with acute pancreatitis in children include autoimmune pancreatitis, Crohn’s disease, diabetes mellitus (diabetic ketoacidosis), Henoch-Schoenlein purpura, and hemolytic uremic syndrome [8, 9]. Therefore, it is important to find the etiological causes of acute pancreatitis for disease management in children.

According to the International Study Group of Pediatric Pancreatitis in Search for a Cure (INSPPIRE) Consortium, diagnosis of pancreatitis in children is defined as having two of the following three signs: 1) abdominal pain associated with pancreatitis; 2) amylase or lipase activity at least three times higher than normal; and 3) imaging findings suggesting pancreatitis. Although contrast-enhanced computed tomography (CT) is the gold standard modality in the diagnosis of acute pancreatitis, ultrasonography is recommended for the initial examination; however, CT scan is not recommended for all patients [10].

The goal of medical treatment for acute pancreatitis is to control pain and maintain metabolic balance. Water, electrolyte, and mineral modification is an important part of patient management [10]. Evidence suggests that an earlier onset of mouth feeding reduces the rate of complications and the length of hospital stay [11]. In severe acute pancreatitis, antibiotics are used to treat infectious necrosis, whereas prophylactic antibiotics are not recommended [9]. Besides, suppression of gastric acid with proton-pump inhibitors may be beneficial [8].

As only few original studies have investigated acute pancreatitis in children in Iran, in the present study, the clinical manifestations and etiological factors of this disease, as well as diagnostic and therapeutic measures, were investigated in children.

## Methods

In this retrospective, cross-sectional study, a total of 60 children with a diagnosis of acute pancreatitis, admitted to Children's Medical Center Hospital in Tehran, Iran, from 2013 to 2020, were studied. The patients’ data were collected by reviewing the hospital data archive. Based on the evaluation of medical records, patients under the age of 18 years, who met the diagnostic criteria for acute pancreatitis according to the INSPPIRE criteria, were included in the study. On the other hand, patients diagnosed with another disease during hospitalization or those without a definite diagnosis of acute pancreatitis were excluded. If a patient had recurrent pancreatitis, only the first occurrence was considered in this study.

The patients’ demographic information, signs, and symptoms were collected from their medical history. To find the predisposing factors for pancreatitis in children, the patients were surveyed for hepatobiliary disease, infectious diseases, systemic diseases, history of trauma, and medication history. Diagnostic data, including laboratory and radiological findings, were also collected and reviewed. The treatment measures were determined based on the physician orders documented in the medical files. All diagnostic measures and therapeutic measures compared with the North American Society for Pediatric Gastroenterology Hepatology and Nutrition Pancreas Committee guideline for management of Acute Pancreatitis in the Pediatric Population [10].

### Statistical analysis methods

Data analysis was performed using SPSS software version 22. The normality of data distribution was assessed using the Shapiro–Wilk test. Mean (standard deviation) and frequency (percentage) were used to describe quantitative and qualitative data, respectively. Independent sample T-Test or, if necessary, its non-parametric equivalent (Mann–Whitney test) was used to compare the mean of quantitative variables. The χ2 test was used for comparisons of categorical variables. In this study, *P* < 0.05 was considered significant. This study was approved by the Tehran University of Medical Sciences (ethics code: IR.TUMS.CHMC.REC.1399.118).

## Results

Among 60 children with acute pancreatitis, 33 were boys (55%), and 27 were girls (45%). The mean age of the patients was 8.25 ± 4.4 years (range: 4 months-16 years). One-third of patients were referred to children’s medical center hospital from other centers throughout Iran.

### Clinical manifestations

Regarding the patients’ clinical manifestations, abdominal pain (91%) and nausea and vomiting (80%) were the most common symptoms. Some patients also had fever, diarrhea, back pain, and jaundice. In the physical examination at the time of admission, the most common sign was epigastric tenderness (50%). Other common signs included tachycardia, tachypnea, and hyperthermia (Table 1).

**Table 1:** Clinical manifestations among patients

*Total number:60*	*Variables*
*55 (91.7%)*	***Abdominal pain***
*48 (80%)*	***Nausea/vomiting***
*30 (50%)*	***Epigastric tenderness***
*19 (31%)*	***Tachycardia***
*15 (25%)*	***Tachypnea***
*9 (15%)*	***Temperature* > *38***
*7 (11.3%)*	***Diarrhea***
*3 (5%)*	***Jaundice***
*3 (5%)*	***Back pain***
*3 (5%)*	***Irritability***

***Values are expressed as number (percentage)***

### Etiologies

The evaluation of predisposing factors for pancreatitis in children showed systemic diseases (21%), drugs (13%), hepatobiliary abnormalities (10%), infectious diseases (10%) and recent trauma history (8%) as common causes of acute pancreatitis. However, the study of 17 (28%) patients did not indicate the specific cause of acute pancreatitis (Table 2). corticosteroid, valproic acid and carbamazepine were the most common causes of drug-induced pancreatitis in this study. All children with acute pancreatitis during infancy had predisposing factors, such as hyperlipidemia.

**Table 2:** The main and secondary etiologies of acute pancreatitis

*Total number:60*	*Variables*			
*6 (10%)*	***Hepato biliary system anomaly***			
*4(6.6%)*	*Choledocal cyst*			
*1 (1.7%)*	*Common bile duct stone*			
*1(1.7%)*	*Pancreatic duct stenosis*			
*6 (10%):*	***Infectious disease***			
*1 (1.7%)*	*Urinary tract infection*			
*1(1.7%)*	*Upper respiratory tract infection*			
*1(1.7%)*	*Spontaneous bacterial peritonitis (SBP)*			
*2 (3.3%)*	*Pneumonia*			
*1(1.7%)*	*Covid-19 infection (positive PCR)*			
*13 (21%)*	***Systemic disease***			
*5 (8.3%)*	*Hyperlipidemia*			
*2 (3.3%)*	*Minor thalassemia*			
*1(1.7%)*	*Systemic lupus erythematosus*			
*1 (1.7%)*	*Hypothyroidism*			
*1(1.7%)*	*End stage renal disease*			
*1 (1.7%)*	*Metabolic disease*			
*1 (1.7%)*	*Cystic fibrosis*			
*1(1.7%)*	*Diabetes mellitus*			
*5(8.3%)*	***Trauma history***			
*3(5%)*	*Blunt trauma*			
*2(3%)*	*Post-surgery*			
*8(13.3%)*	***Drugs associated with acute pancreatitis***			
*3 (5%)*	*Sodium valproate*			
*4(6.6%)*	*Corticosteroid*			
*4(6.6%)*	*Carbamazepine* + *sodium valproate*			

***Values are expressed as number (percentage)***

The North American Society for Pediatric Gastroenterology Hepatology and Nutrition Pancreas Committee, has published a guideline for management of Acute Pancreatitis in the Pediatric Population in 2018.(9) In this study all diagnostic measures and therapeutic measures compared with this guideline to determine whether our pediatricians follow the guideline recommendations or not.

### Diagnostic measures

Elevated serum amylase and lipase activity was the most common finding in the tests. The lowest level of amylase was 13 U/L, while its highest level was 6,117 U/L. Besides, the lowest lipase level was 20 U/L, while the highest level was 4,510 U/L. Normal amylase and lipase levels were observed in only four patients with hyperlipidemia during medical assessments. In addition, increased inflammatory markers and leukocytosis were common findings in the patients. Elevated hepatic transaminases and increased levels of gamma-glutamyl transferase (GGT) were also observed in some patients. Hypocalcemia, hyperglycemia, and hypoglycemia were detected in a small number of patients. Also, regarding the patients’ lipid profile, four patients were diagnosed with hyperlipidemia (Table 3). Considering the imaging procedures, ultrasonography was performed for 53 (88%) patients; findings consistent with pancreatitis were found in only 27 (50%) cases. Moreover, CT scan was performed for 22 (36%) patients, 17 (77%) of whom showed evidence of acute pancreatitis. In the clinical case study of 7 (31%) patients, for whom a CT scan was performed, no specific indication was found for CT scan, based on the medical records (Table 3).

**Table 3:** Imaging and Laboratory data analysis obtained from patients with acute pancreatitis

Total number: 60 patients	Variables	
	**Abdominal sonogaraphy**	
53(88%)	Performed	
27(45%)	Positive for acute ancreatitis	
	**Abdominal computed tomography(CT scan)**	
22 (36%)	Performed	
17(28%)	Positive for acute ancreatitis	
7(11.6%)	No clear indication for CT scan	
	**Magnetic resonance cholangiopancreatography (MRCP)**	
5 (8%)	Performed	
32 (53%)	**Leukocytosis**	
10 (16.6%)	**Anemia**	
8 (13%)	**Thrombocytosis**	
7 (11.6%)	**tThrombocytopenia**	
2 (3.3%)	**Abnormal renal function test**	
2 (3.3%)	**Hypoglycemia**	
5 (8.3%)	**Hypergycemia**	
3 (5%)	**Hypocalcemia**	
54 (90%)	**Raised amylase**	
55 (91%)	**Raised lipase**	
4 (6.6%)	**Hyperlipidemia**	
8 (13.3%)	**Raised transaminases**	
6 (10%)	**Raised gamma GT**	
6 (10%)	**Raised ALK P**	
15 (25%)	**Raised ESR/CRP**	

***Values are expressed as number (percentage)***

### Therapeutic measures

The therapeutic measures taken for the patients were examined in this study (Table 4). For 11 (18%) patients, an isotonic intravenous bolus fluid was prescribed to treat dehydration. Only half of the patients received fluid 1.5 times their maintenance in the first 24 h. Analgesia was prescribed for only 28 (46%) patients. Fifteen patients (25%) kept NPO for more than 24 h. Antibiotic therapy was performed for 28 (48%) patients. Medical indications for antibiotic treatment (e.g., infectious necrosis) were found in only 10 (34%) cases, based on the medical records. One of the patients initially underwent surgery due to a diagnosis of acute appendicitis; however, during surgery, it was found that the appendix was normal and that the patient had acute pancreatitis.

**Table 4:** Therapeutic measures performed among patients with acute pancreatitis

Total number:60	Therapeutic measures	
11(18%)	**Resuscitation fluid**	
30(50%)	Correct fluid therapy (1.5–2* maintenance)	
28 (46%)	**Analgesic treatment**	
16(26%)	**Acetaminophen**	
12(20%)	**Petedine**	
54(90%)	**Proton pomp inhibitor**	
	**Start oral feeding**:	
20 (33%) 25 (41%) 15 (25%)	0-12 h 12–24 h later than 24 h	
29 (48%)	**Antibiotic**	
19(31.6%)	No clear indication for antibiotic therapy	
1 (1.6%)	**TPN**	
4 (6%)	**Surgery**	

***Values are expressed as number (percentage)***

### Outcomes

Four patients required surgery after being diagnosed with acute pancreatitis. The causes of surgery were choledochal cyst excision, pancreatic pseudocysts, and pancreatic lesions due to trauma, followed by recurrent and divisum pancreatitis (Table 4).

One patient with a history of cerebral palsy, presenting with fever, seizures, sepsis, and acute pancreatitis, expired after 15 days of intensive care unit (ICU) admission. It should be noted that in the last six months of this study (i.e., the first half of 2020), the COVID-19 pandemic occurred. During these six months, 11 cases of acute pancreatitis were documented in our hospital. Four cases had clinical, imaging, and laboratory evidence of COVID-19 and were admitted to the COVID-19 ward; one of them had positive nasopharyngeal PCR results for COVID-19.

## Discussion

In the present study, which was done on 60 children with acute pancreatitis, the number of boys (55%) was slightly higher than girls. In previous studies, the male-to-female ratio ranged from 0.9 to 1.2, which is not significantly different from the present study [2, 12–15]. The mean age of children was 8.2 years; also, other studies reported an age range of 6.9–12.5 years [2, 12–15]. In the present study, the most common clinical symptom was abdominal pain in the epigastric region (91%), followed by nausea and vomiting (80%). In other previous studies, abdominal pain (95–80%) was also the most common clinical symptom [10, 16, 17].

Importantly, the clinical signs of acute pancreatitis may be atypical during infancy (e.g., nausea and vomiting, fever, irritability, and abdominal distension) [18]. Therefore, it is essential to consider the diagnosis of acute pancreatitis, especially in infants with the above-mentioned unexplained symptoms. In this study, the most common causes of acute pancreatitis were systemic and metabolic diseases (35%), medications (13%), hepatobiliary diseases (10%), and infections (10%). In other studies, systemic diseases, especially diabetic ketoacidosis, sepsis, and hemolytic uremic syndrome, were the etiologies of 10% to 50% of cases [10, 16, 17]. In the present study, the most common systemic diseases associated with pancreatitis was hyperlipidemia.

In previous studies, medications were the cause of 10% to 30% of acute pancreatitis cases [10, 16–18]. The most common medications reported in previous studies were valproic acid, L-asparagine, 6-mercaptopurine, azathioprine, steroids, mesalamine, and metronidazole [10, 15, 16, 19]. Additionally, the most common biliary cause of acute pancreatitis in the present study was choledochal cyst, while in other studies, pancreas divisum, biliary stones, and biliary stenosis were the most common anatomical causes [10, 16, 17]; the delayed diagnosis of choledochal cysts in Iran may be the cause of this difference. Corticosteroid, valproic acid, and carbamazepine were the most common causes of drug-induced pancreatitis in this study.

In the present study, no specific cause was found for acute pancreatitis in 28% of cases. The corresponding rate was estimated at 20–24% in similar studies [10, 16, 17]; this slight difference might be due to limitations in the diagnostic accuracy of modalities. Ultrasonography was performed in 88% of patients in our study; in half of the cases, the findings were consistent with acute pancreatitis. Although ultrasound is recommended as the first diagnostic method for the diagnosis of acute pancreatitis, it is operator-dependent [10, 18, 19]. An abdominal CT scan was performed for more than one-third of cases in this study. According to guidelines the most important indications for CT scan were necrosis, abscess, and other complications. Performing CT scan is not indicated nor obligatory for diagnosis or management of acute pancreatitis in children. In cases that are vague for a diagnosis, a CT scan may be needed to confirm acute pancreatitis. [10, 18, 19]. Given the risk of radiation exposure, the need to perform a CT scan must be carefully considered. Also, in one-third of CT scans performed in this study, no specific reason was found for the CT scans, based on the hospital records.

Adequate fluid therapy (Bolus fluid infusion with 10–20 cc/kg normal saline or lactate ringer and intravenous fluid therapy with 1.5–2 times maintenance in the first 24–48 h of disease are recommended according to guidelines.) and early onset of oral nutrition play important roles in reducing the length of hospitalization and the need for ICU admission and decreasing the likelihood of disease progression into its severe form [10, 18, 19]. Only 18% of patients in the current study received bolus intravenous fluid therapy, and only half of the patients obtained more fluid than the maintenance requirement in the first 48 h of disease; therefore, fluid therapy should be emphasized in clinical education due to its great importance in these patients.

Moreover, one-quarter of patients remained NPO after the first 24 h, which also increased the need for further training of physicians. Almost half of the patients in this study were treated with antibiotics, and according to the hospital records, only one-third of them had indications for antibiotic treatment. According to the North American Society for Pediatric Gastroenterology Hepatology and Nutrition Pancreas Committee guidelines, administration of antibiotics for acute pancreatitis is only necessary in the case of infectious necrosis or disease progression into necrotic pancreatitis [10]. Besides, the inappropriate use of antibiotics, along with the side effects of medications, increases the likelihood of antibiotic resistance. In this study, only one child with cerebral palsy expired, with signs of sepsis, seizures, and acute pancreatitis. According to previous studies, the mortality rate of acute pancreatitis in children is less than 5%, which is consistent with our study [10, 16, 17].

The relatively increased incidence of acute pancreatitis in our center during the COVID-19 pandemic may be related to this infection; before the pandemic, 4–5 cases of acute pancreatitis were registered every six months according to the hospital records, while after the pandemic in Iran, 11 cases were registered during six months. It seems that several viruses play an etiological role in acute pancreatitis, including mumps, measles, Epstein-Barr virus (EBV), hepatitis A virus (HAV), hepatitis E virus (HEV), and coxsackievirus [20, 21]; therefore, an association between SARS-CoV-2 and pancreatitis is probable.

Previous studies have confirmed the gastrointestinal involvement of SARS-CoV-2 [22]. However, limited case reports have been published on the association between COVID-19 and acute pancreatitis in adult or pediatric populations during this pandemic [23–25]. The proposed pathophysiology of pancreatic involvement in COVID-19 is the expression of angiotensin-converting enzyme 2 (ACE2) in both islet cells and the exocrine portion of the pancreas [26, 27]. Pancreatic injury during an acute SARS-CoV-2 infection can be also related to indirect systemic inflammatory and immune-mediated cellular responses. Besides, antipyretics that are commonly used for COVID-19 patients can cause drug-related pancreatic damages [26, 27]. Further research is required to determine the definite effects of SARS-CoV-2 on pancreatic function and regulation.

## Conclusion

Considering the increasing prevalence of acute pancreatitis in children, if symptoms, such as vomiting or abdominal pain, occur in a patient with systemic or metabolic diseases, acute pancreatitis should be considered. Medications play an important role in the development of acute pancreatitis. In children using anticonvulsants, immunosuppressant drugs, and chemotherapy, it is important to consider the incidence of this disease. Moreover, in clinical education, the risks of radiation due to unnecessary CT scans for children with acute pancreatitis need to be emphasized. Besides, inappropriate prescription of antibiotics, insufficient fluid therapy, and delayed initiation of oral feeding in these patients should be highlighted in therapeutic and educational programs. Also, healthcare providers should consider COVID-19 as a differential diagnosis when managing patients with gastrointestinal symptoms.

## Data Availability

The datasets used and/or analysed during the current study are available from the corresponding author on reasonable request.
