# Correction: Acute pancreatitis in 60 Iranian children: do pediatricians follow the new guidelines in diagnosis and management of acute pancreatitis?

**DOI:** 10.1186/s12887-022-03538-1

**Published:** 2022-08-22

**Authors:** Mahsa Soti Khiabani, Mahya Sadat Mohammadi, Seyyed Amirreza Ghoreyshi, Pejman Rohani, Hosein Alimadadi, Mohammad Hassan Sohouli

**Affiliations:** 1 Department of Pediatrics, Tehran University of Medical Sciences, Tehran, Iran; 2 Pediatric Gastroenterology and Hepatology Research Center, Tehran University of Medical Sciences, Tehran, Iran; 3 Children’s Medical Center, Tehran University of Medical Sciences, Tehran, Iran; 4 Student Research Committee, Department of Clinical Nutrition and Dietetics, Faculty of Nutrition and Food Technology, Shahid Beheshti University of Medical Sciences, Tehran, Iran

**Correction: BMC Pediatr 22, 457 (2022)**

**https://doi.org/10.1186/s12887-022-03509-6**

Following publication of the original article [1], the authors identified an error in two of the author names: the letter ‘i’ at the end of the names of "Hosein AliMadadi" and "Mohammad Hassan Sehouli" had been mistakenly removed, and the Acknowledgements declaration had been incorrectly written (namely, the department that Dr Pejman Rohani heads had been incorrectly written).

The original article has been corrected. Moreover, the corrected author names can be found in the author list of this erratum and, likewise, the corrected Acknowledgements is available below.

**Acknowledgements**

This study is taken from the Recurrent Acute Pancreatitis and Chronic Pancreatitis in Pediatrics registry program at Tehran University of Medical Sciences (IR.TUMS.MEDICINE.REC.1400.1527). We are thankful to Dr Pejman Rohani, head of the Pediatrics Recurrent Acute Pancreatitis and Chronic Pancreatitis registry at Tehran University of Medical Sciences.
